# Evaluation of diastolic function: machine learning improves classification of left ventricular filling pressure

**DOI:** 10.1093/ehjci/jeag025

**Published:** 2026-01-28

**Authors:** Faraz H Khan, Katsuji Inoue, Nobuyuki Ohte, Eusebio García-Izquierdo, Michael Chetrit, Vanessa Moñivas-Palomero, Susana Mingo-Santos, Øyvind S Andersen, Einar Gude, Kaspar Broch, Tom Kai Ming Wang, Shohei Kikuchi, Jong-Won Ha, Allan L Klein, Sherif F Nagueh, Otto A Smiseth, Espen W Remme

**Affiliations:** Institute for Surgical Research and Department of Cardiology, Oslo University Hospital, Rikshospitalet, and University of Oslo, Sognsvannsveien 20, 0372, Oslo, Norway; Institute for Surgical Research and Department of Cardiology, Oslo University Hospital, Rikshospitalet, and University of Oslo, Sognsvannsveien 20, 0372, Oslo, Norway; Department of Cardiology, Pulmonology, Hypertension & Nephrology, Ehime University Graduate School of Medicine, Toon, Japan; Department of Cardiology, Nagoya City University Graduate School of Medical Sciences, Nagoya, Japan; Cardiology Unit, Hospital Universitario Puerta de Hierro Majadahonda, Madrid, Spain; Department of Cardiovascular medicine, Heart, Vascular and Thoracic institute, Cleveland Clinic, Cleveland, OH, USA; Cardiology Unit, Hospital Universitario Puerta de Hierro Majadahonda, Madrid, Spain; Cardiology Unit, Hospital Universitario Puerta de Hierro Majadahonda, Madrid, Spain; Institute for Surgical Research and Department of Cardiology, Oslo University Hospital, Rikshospitalet, and University of Oslo, Sognsvannsveien 20, 0372, Oslo, Norway; Institute for Surgical Research and Department of Cardiology, Oslo University Hospital, Rikshospitalet, and University of Oslo, Sognsvannsveien 20, 0372, Oslo, Norway; Institute for Surgical Research and Department of Cardiology, Oslo University Hospital, Rikshospitalet, and University of Oslo, Sognsvannsveien 20, 0372, Oslo, Norway; Department of Cardiovascular medicine, Heart, Vascular and Thoracic institute, Cleveland Clinic, Cleveland, OH, USA; Department of Cardiology, Nagoya City University Graduate School of Medical Sciences, Nagoya, Japan; Cardiology Division, Yonsei University College of Medicine, Seoul, Korea; Department of Cardiovascular medicine, Heart, Vascular and Thoracic institute, Cleveland Clinic, Cleveland, OH, USA; Methodist DeBakey Heart and Vascular Center, Houston Methodist Hospital, Houston, TX, USA; Institute for Surgical Research and Department of Cardiology, Oslo University Hospital, Rikshospitalet, and University of Oslo, Sognsvannsveien 20, 0372, Oslo, Norway; Institute for Surgical Research and Department of Cardiology, Oslo University Hospital, Rikshospitalet, and University of Oslo, Sognsvannsveien 20, 0372, Oslo, Norway; The Intervention Centre, Oslo University Hospital, Rikshospitalet, Oslo, Sognsvannsveien 20, 0372, Oslo, Norway

**Keywords:** machine learning, diastolic dysfunction, left ventricular filling pressure, echocardiography, heart failure, heart catheterization

## Abstract

**Aims:**

Current recommendations for echocardiography-based classification of left ventricular filling pressure (LVFP) as normal or elevated, are based on an algorithm and parameter selection determined by human experts. We tested whether machine learning (ML) can improve classification of LVFP and investigated which parameters were deemed most important by different ML models.

**Methods and results:**

In a multicentre study, echocardiography was performed simultaneously with, or within 24 h of, heart catheterization in 250 patients. Eight different ML models were trained and tested using a nested cross-validation procedure to classify LVFP as normal or elevated. The training included a search and selection of the most useful parameters. Performance was assessed from the test sets not seen during training. The eight ML models could classify all patients regardless of missing parameter values with accuracy ranging from 82% to 86%. The 2016 ASE/EACVI guidelines algorithm left 13% unclassified due to missing values and had an accuracy of 81% in the remaining patients. On average, the eight ML models selected 13 parameters, and left atrial strain was included in three of these. The five highest ranked parameters by the ML models were mitral E/left atrial reservoir strain, log(NT-proBNP), tricuspid regurgitation velocity, septal E/e’, and E/A.

**Conclusion:**

ML can improve classification of LVFP, particularly with a higher feasibility. The study unveiled less used parameters as some of the most valuable for evaluating LVFP.

## Graphical Abstract

**Graphical Abstract:**
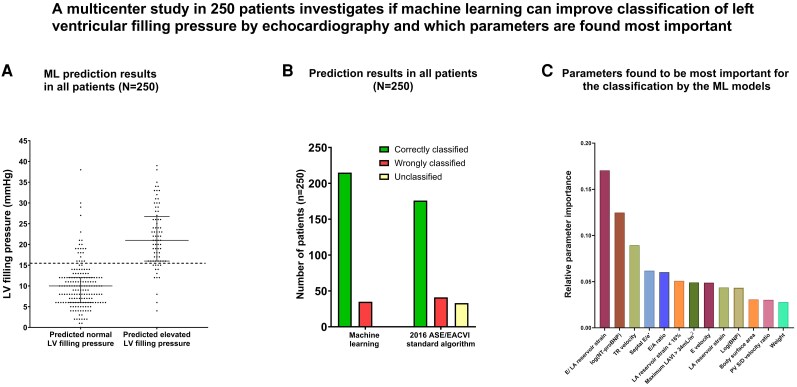
Classification of left ventricular filling pressure by machine learning. Panel A shows the classification prediction (x-axis) from the logistic regression machine learning classifier in all 250 patients relative to the invasively measured LVFP (y-axis). The horizontal bars represent mean pressure ± standard deviation. Panel B shows the classification performance of the machine learning classifier compared with the 2016 ASE/EACVI algorithm. Panel C shows which parameters the investigated machine learning models found to be most useful in the classification process. The 13 most important parameters are shown, ranked according to their relative parameter importance score.

**See the editorial comment for this article ‘Artificial intelligence and the left atrial filling index (mitral E/LA strain ratio) as novel tools for the evaluation of left ventricular diastolic dysfunction: an editorial commentary and systematic review’, by D.A. Morris, https://doi.org/10.1093/ehjci/jeag073.**

## Introduction

Evaluation of left ventricular (LV) filling pressure (LVFP) is an integral part of the diagnostic work-up in patients suspected of heart failure (HF). Heart catheterization is the gold standard for evaluating LVFP but is performed in only a minority of patients due to invasiveness and costs. Echocardiography is the most widely used non-invasive method for assessment of LVFP. Despite numerous studies, no single echocardiographic parameter has been shown to robustly classify LVFP as normal or elevated. In 2016 the European association of cardiovascular imaging (EACVI) and the American society of echocardiography (ASE) published a joint consensus document which recommended a combination of echocardiographic parameters for assessment of LVFP.^1^ Two large multicentre studies comparing invasive measurements of LVFP with echocardiographic parameters have confirmed the validity of these recommendations, but they were limited by relatively large fractions of unclassified patients due to missing parameters.^2,3^ This points to a weakness of the recommendations which is that several patients are left unclassified due to difficulties with measuring all parameters. In a large multicentre study on echocardiographic evaluation of LVFP, we showed that replacing a missing parameter in the 2016 ASE/EACVI algorithm with LA strain reduced the number of unclassified patients.^4^ This addition of LA reservoir strain to the algorithm was recommended in the recent 2022 EACVI consensus document on how to evaluate LVFP in patients with heart failure and preserved ejection fraction.^5^ The 2016 and 2022 algorithm is a so-called decision tree with a small number of parameters selected by human experts based on results from previous studies and also multicentre testing^4^ in the 2022 case.

As an alternative to a human derived algorithm, development of such a classification model seems a highly suitable task for machine learning (ML). We suggest that ML with testing of an extended number of diagnostic parameters may allow development of better classification models than the current algorithms. ML uses mathematical techniques to find the optimal algorithm with the best combination of parameters and cutoff values. Recently, the group led by Sengupta used a deep learning approach to predict high- and low-risk groups of diastolic dysfunction.^6^ Their model was thus not developed specifically for classification of LVFP. However, when applied on 84 patients with invasively measured LVFP, the method had a higher area under the curve (AUC) than the 2016 ASE/EACVI algorithm.

In this study, we aimed to develop and test ML models specifically for classifying LVFP based on a dataset of 250 patients with invasively measured pressures. The ML models were developed to handle cases with missing parameters. The performance of the method was evaluated and compared to the 2016 ASE/EACVI algorithm as well as the 2022 EACVI algorithm^5^ and single parameters. Finally, we investigated which parameters the ML approach found most useful for classifying LVFP.

## Methods

### Data collection

Patients referred for diagnostic right- or left-sided heart catheterization for suspected heart failure, cardiomyopathy, coronary artery disease or pulmonary hypertension were enrolled at Oslo University Hospital, Rikshospitalet (Oslo, Norway), Methodist DeBakey Heart and Vascular centre (Houston, TX, USA), Yonsei University College of Medicine (Seoul, South-Korea), Cleveland Clinic (Cleveland, OH, USA), Hospital Universitario Puerta de Hierro, Majadahonda (Madrid, Spain), and Nagoya City University, Graduate School of Medical Sciences (Nagoya, Japan). Patients were included from January 2014 until December 2023. Patients with complex congenital heart disease, heart transplants, end-stage liver disease, mitral stenosis, or prosthetic mitral valve were excluded. Furthermore, we excluded 158 patients with atrial fibrillation, left bundle branch block, paced rhythm, non-cardiac pulmonary hypertension, restrictive or hypertrophic cardiomyopathy, and mitral regurgitation of at least moderate grade, as such pathologies typically alter the relation between echocardiographic parameters and LVFP and should be evaluated by dedicated algorithms.^1^

After exclusion, the data consisted of 250 patients. This included 196 patients from our previous multicentre study^4^ and 54 additional patients, where 108 and 142 were prospectively and retrospectively included, respectively.

The study was approved by the Regional Ethics Committees and Institutional Review Boards at all participating centres.

### Echocardiographic imaging

Different echocardiography equipment was used at the participating centres, including GE Healthcare (Echopac), Philips (QLAB), and Siemens (VVI, Tomtec). Echocardiographic examination was performed either during catheterization or maximum within 24 h. The imaging and analyses were performed by experienced investigators who were blinded to the invasive measurements. Parameters were averaged over three cardiac cycles. A total of 64 different parameters were included, which in addition to echocardiographic parameters included age, sex, height, weight, comorbidities, arterial blood pressure, as well as proBNP and creatinine when available (see Supplementary data online, *Figure S1*).

### Cardiac catheterization

In 187 patients, LVFP was measured during right heart catheterization as end-expiratory pulmonary arterial wedge pressure. In 63 patients, LVFP was measured during left heart catheterization as LV pre-atrial contraction (pre-A) pressure (*n* = 23), and when pre-A pressure was not possible to assess, as LV end-diastolic pressure (*n* = 40). Pulmonary arterial wedge pressure or LV pre-A pressure >15 mmHg, or LV end-diastolic pressure >16 mmHg was considered elevated.

### Machine learning

We evaluated eight different ML classifiers based on different mathematical algorithms:

- Extreme gradient boosting (XGBoost)
- Support vector classifier
- Linear support vector classifier
- Gradient boosting classifier
- Logistic regression classifier
- K-nearest neighbours classifier
- Random forest classifier
- Decision tree classifier

In contrast to the seven others, the XGBoost model handles missing parameters internally and thus allowed classification of all patients. For the other seven, missing parameters were substituted to prevent many unclassified patients. To make the effect of a missing parameter as neutral as possible on the model prediction, it was substituted with the value considered to classify LVFP as normal or elevated with a 50–50% probability, i.e. its cutoff value. This cutoff value was found from logistic regression of each parameter as a single predictor. For example, for average a’, 5.1 cm/s was found as the best cutoff to differentiate LVFP and hence used to substitute missing a’ values (see Supplementary data online, *Figure S1*, third panel).

A secondary purpose of this study was to investigate which parameters the ML models would find most useful for classifying LVFP. As many of the registered parameters had no significant correlation to LVFP and not all parameters could be obtained for every patient, an initial parameter selection was performed to reduce the number of candidate parameters to be considered by the ML model. Supplementary data online, *Figure S1* shows regression plots between LVFP and the 64 parameters that we considered initially. All parameters with a significant correlation coefficient with LVFP that were present in 80% or more of the patients, were selected. However, several of the best correlated parameters were available in less than 80% of the patients, and these could potentially improve classification when present. Based on visual inspection of the regression plots in Supplementary data online, *Figure S1*, we therefore chose also to select the parameters with absolute correlation coefficient higher than 0.40 that were present in less than 80% of the patients. The described selection process reduced the original 64 parameters to 47 candidate parameters which were passed to the ML models for further search for the optimal parameters as explained below.

### Training and testing the ML models

**Figure 1:**
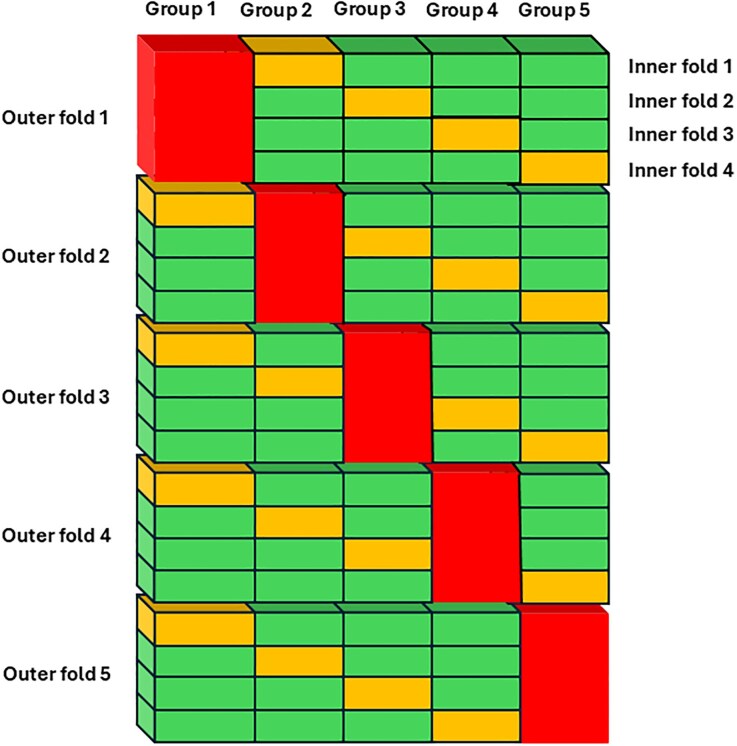
The nested cross-validation procedure. The patients were randomly divided into 5 groups. The red group illustrates the group that was left out for testing. The 4 other groups were used to train the machine learning model using an inner 4-fold cross-validation where yellow indicates the validation group and the patients in the green groups were used for training. Four models were thus trained in this inner 4-fold cross-validation, for example to search for the optimal parameter set. The parameter set with the best average score on the 4 validation groups, were selected. The output from the internal 4-fold cross-validation was a single model trained using the data from all four groups with the selected parameters. This model was then tested on the red group. The procedure was repeated five times leaving a different group out for testing each time. The performance of the machine learning method was evaluated as the average of the five test groups.

The data from the 250 patients was split into training and test sets. We employed a nested cross-validation approach to ensure robust and unbiased evaluation of the ML models. First, the dataset was randomly divided into five groups with similar relative distributions of patients with normal and elevated LVFP. As shown in *Figure 1*, one of the five groups were left out for testing and not ‘seen’ during training of the model. The remaining four groups were used to train the model employing a 4-fold cross-validation where the first step was finding the optimal parameters out of the 47 candidate parameters using the recursive feature elimination cross-validation method (scikit-learn 1.3.0). Once the optimal parameters had been found, the final training step was optimizing the hyperparameters of the ML model using a full grid search. The Supplemental methods describe the hyperparameter search in detail.

This nested cross-validation procedure was performed five times, leaving a different group out for testing each time, and training on the remaining 4 groups. The average performance on the 5 different test sets was considered the performance of the model on unseen data.

### Evaluation of performance

The accuracy, receiver operating characteristic AUC, sensitivity, specificity, positive, and negative predictive values for correct classification of the LVFP were extracted. The ML models left no patient unclassified. For the 2016 ASE/EACVI algorithm as well as the 2022 EACVI algorithm and single parameters, these performance metrics were evaluated for the classified patients only, and the percentage of unclassified patients was also presented. The single parameters evaluated were: E/e′ > 14, tricuspid regurgitation velocity > 2.8 m/s, maximum left atrial volume index > 34 mL/m², E/A < 0.8, E/A > 2, LA reservoir strain < 23%, LA reservoir strain < 18%, mitral E/LA reservoir strain > 3, and mitral E/LA reservoir strain > 5.

### Parameter importance

For each ML model, the feature elimination cross-validation method found the optimal set of parameters for each model. Four of the ML models also ranked the selected parameters according to their usefulness in the ML-model with a ratio that summed up to 1. The four other models selected parameters without providing ranking. However, we weighted their selected parameters equally so that if a model selected 10 parameters, we assigned 0.1 to each and zero to the non-selected parameters. We subsequently averaged these ratio-scores for each parameter from the eight models, which then allowed an overall ranking of the most important parameters found in the ML models. This analysis of parameter importance was conducted using the entire dataset of 250 patients, employing 5-fold cross-validation.

### Statistics

Patient characteristics are presented as median (interquartile range) or number (percentage). Linear regression analysis was used to assess correlation between variables. AUC values for the 2016 ASE/EACVI algorithm, the 2022 EACVI algorithm and single parameters were calculated from the sensitivity and specificity values. A two-tailed *P* < 0.05 was considered significant for all statistical analyses. Statistical analyses were performed using Graphpad prism version 10 and Python version 3.11.4.

## Results

### Clinical, haemodynamic, and echocardiographic data

*Table 1* summarizes the clinical, haemodynamic, and echocardiographic data. LV ejection fraction (EF) was ≥50% in 69%. LVFP was elevated in 34%.

**Table 1:** Clinical, haemodynamic, and echocardiographic characteristics

Variables	Median (interquartile range) or number (percentage)
**Clinical**	
Age, years	62 (54–70)
Female gender, *n* (%)	110 (44)
Body mass index, kg/m^2^	26 (23–29)
Hypertension, *n* (%)	106 (56)
Chronic kidney disease, *n* (%)	38 (3)
**Haemodynamic**	
Heart rate, beats/min	70 (60–79)
Systolic blood pressure, mmHg	123 (111–142)
Diastolic blood pressure, mmHg	70 (62–79)
PCWP, mmHg	12 (7–21)
LV pre-A, mmHg	10 (7–12)
LV end-diastolic pressure, mmHg	13 (10–17)
**Echocardiography**	
LV end-diastolic volume, mL	101 (79–133)
LV end-systolic volume, mL	39 (27–68)
LV ejection fraction, %	58 (44–66)
E velocity, cm/s	72 (58–91)
A velocity, cm/s	73 (54–90)
E/A	1.0 (0.8–1.4)
Average E/e’	11 (8–15)
Peak TR velocity, m/s	2.6 (2.2–3.0)
maxLAVI, mL/m^2^	34 (26–43)
LV GLS, %	15 (11–18)
LA reservoir strain, %	21 (12–29)
LA pump strain, %	9 (5–14)

GLS, global longitudinal strain; LA, left atrial; LV, left ventricular; maxLAVI, maximum LA volume index; PCWP, pulmonary arterial wedge pressure; TR, tricuspid regurgitation.

### Classification performance

The average performance from the five test sets showed that the eight ML models had an accuracy in the range 82% to 86% for evaluating LVFP correctly as normal or elevated, and seven of the models had an AUC in the range 0.84 to 0.89. The decision tree classifier, which was the simplest of the eight ML models, had AUC = 0.80. *Table 2* and panel A and B in *Graphical Abstract* show the performance of the Logistic regression classifier which had accuracy = 86% and AUC = 0.87. Supplementary data online, *Table S1* shows the detailed performance of each model.

**Table 2:** Performance of the logistic regression machine learning (ML) classifier, the 2016 ASE/EACVI algorithm, the 2022 EACVI algorithm and single parameters in all 250 patients where 34.4% had elevated left ventricular filling pressure

	Accuracy (%)	AUC	Sensitivity (%)	Specificity (%)	PPV (%)	NPV (%)	Unclassified (%)
ML classifier	86	0.87	75	92	84	87	0
2016 ASE/EACVI	81	0.80	74	85	73	86	13
2022 EACVI	78	0.76	68	84	69	83	3
E/e’>14	71	0.66	49	83	61	75	8
TRV > 2.8 m/s	74	0.71	60	83	68	77	24
LAVI > 34 mL/m²	64	0.65	69	62	50	78	6
E/A < 0.8	48	0.58	82	33	36	80	6
E/A > 2	76	0.64	32	96	79	76	6
LARS < 23%	61	0.63	72	54	47	78	8
LARS < 18%	73	0.71	65	78	62	80	8
E/LARS > 3	66	0.70	81	58	52	85	9
E/LARS > 5	78	0.74	60	87	72	80	9

ASE, American society of echocardiography; AUC, area under the curve; EACVI, European association of cardiovascular imaging; EF, ejection fraction; LARS, left atrial reservoir strain; LAVI, maximum left atrial volume index; NPV, negative predictive value; PPV, positive predictive value; TRV, tricuspid regurgitation velocity.

The 2016 ASE/EACVI algorithm left 13% of the patients unclassified due to missing parameter values. The accuracy in the classified patients was 81% and AUC 0.80. (*Table 2* and *Graphical Abstract*, panel B). The 2022 EACVI algorithm left only 3% unclassified. The accuracy in the classified patients was 78% and AUC 0.76. (*Table 2*). *Table 2* also shows the performance of the single parameters. Supplementary data online, *Figure S1* shows correlations between each of the initial 64 parameters and LVFP, and the plots include the number of patients with each parameter and standalone accuracy and AUC using optimal cutoff values found using logistic regression.

The performance of the ML models, manual algorithms and selected single parameters was also assessed in the subpopulations with EF ≥ 50%, EF < 50% and EF < 40%. Supplementary data online, *Table S2*-S4 show the details of this assessment. Generally, performance was poorer in the preserved EF group. and vice versa for the reduced EF groups. There were 173 patients with EF ≥ 50%, and 34% of these had elevated LVFP. While specificity was ≥90% for the ML models, sensitivity was poor and <50% for most models. There were 77 patients with EF < 50%, and 53% of these had elevated LVFP. Most of the ML models had sensitivity and specificity >90% in this group. In the 55 patients with EF < 40%, 67% had elevated LVFP. Sensitivity remained high while specificity was somewhat lower.

### Parameter importance

Of the 47 candidate parameters, 13 were selected on average by the eight ML models. The average estimated relative importance for the top 13 parameters is shown in *Graphical Abstract*, panel C. The ratio mitral E/LA reservoir strain was ranked the most important parameter followed by log(NT-proBNP), tricuspid valve regurgitation velocity, septal E/e’ and E/A.

## Discussion

In this study, we trained and tested eight ML models to classify LVFP primarily based on echocardiographic parameters, using invasively measured pressure as reference. The results showed that the ML models had slightly better accuracy than the 2016 ASE/EACVI algorithm. A main advantage of the ML models was that all patients could be classified. In contrast, 13% were unclassified by the 2016 ASE/EACVI algorithm due to missing parameters. The 2022 EACVI algorithm had only 3% unclassified and was slightly less accurate.

Manual selection of parameters that should be included in the recommendations for classification of LVFP, is challenging. Some parameters that correlate well, such as proBNP or pulmonary venous flow velocities, may be less commonly obtained. A manual method, such as the 2016 ASE/EACVI algorithm, must have a limited number of parameters to be manageable. Hence, rare but good parameters may be left out. In contrast, an ML model is not limited by the memory of the operator. Furthermore, missing parameters can be handled. Hence, if a parameter is well-correlated with LVFP, but available in few patients, it may still be included in the classification when present.

**Figure 2:**
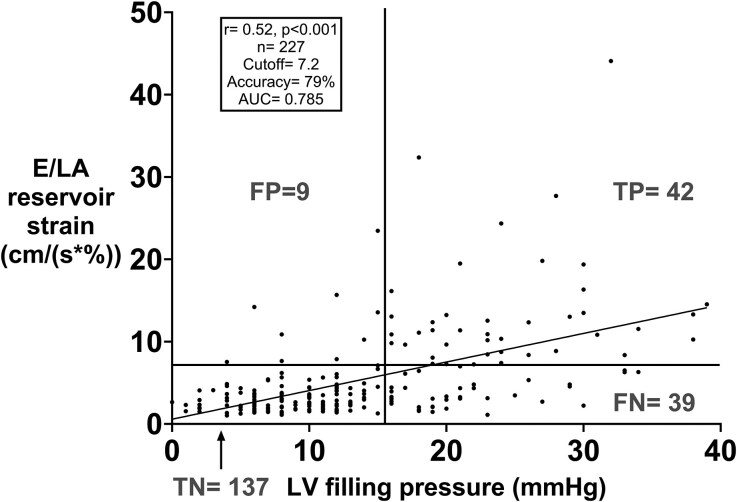
Mitral E/LA reservoir strain vs. LV filling pressure regression plot. The optimal cutoff for classifying LV filling pressure found according to logistic regression is shown together with the corresponding classification accuracy and AUC. The true positive (TP), true negative (TN), false positive (FP), and false negative (FN) classified patients are indicated in their respective quadrants.

An interesting result of our study was which parameters the ML models regarded as the most important parameters as shown in the *Graphical Abstract* (panel C). The ratio mitral E/LA reservoir strain was the most important parameter. This ratio which was previously proposed as marker of LV filling pressure,^7^ combines mitral E which reflects the transmitral pressure difference, and LA reservoir strain, which is related to minimum LV and LA pressures.^8^ From the regression plot of mitral E/LA reservoir strain vs. LVFP in *Figure 2*, it can be seen that a high value of this ratio implies a high probability for elevated LVFP as there would be few false positives, while low value implies a probability of normal LVFP, though with some more false negatives. Since high values of mitral E are observed not only when LA pressure is elevated but also when minimum LV pressure is low or even negative, which is typical for healthy young subjects, elevated mitral E is not a specific marker of elevated filling pressure. Since low values of LA reservoir strain indicate elevated LV minimum pressure,^8^ the combination of high E and low LA reservoir strain is consistent with elevated LA pressure.

Log(NT-proBNP) was ranked second, and log(BNP) was ranked tenth by the ML models, which is consistent with these parameters being two of the best correlated with LVFP (see Supplementary data online, *Figure S1*). However, these natriuretic peptide parameters are less frequently obtained. The conventional parameters tricuspid regurgitation velocity, septal E/e’ and E/A, were ranked third to fifth, respectively, and maximum left atrial volume index > 34 mL/m^2^ as seventh.

Measuring LA strain seems important for classification of LVFP. LA strain was included in three of the top selected parameters by the ML models (*Graphical Abstract*, panel C): first as part of the mitral E/LA reservoir strain ratio, second as the dichotomous parameter LA reservoir strain < 16% and third as peak LA reservoir strain on its own. This is consistent with the growing support for LA strain as a non-invasive marker of LV filling pressure as well as for evaluating diastolic dysfunction using traditional algorithms or ML models.^4,9–11^ Notably, the LA strain waveform may provide useful information other than just peak LA reservoir strain.^12^ LA pump strain > 14% seems associated with normal LVFP.^5^ Another study showed that using ML to weight different timepoints along the LA strain waveform, could be used to extract an index that had at least similar classification accuracy as the 2016 ASE/EACVI algorithm with fewer unclassified patients.^9^ According to the ASE/EACVI consensus report, LA strain should be assessed from apical 4-chamber view.^13^

Pulmonary venous S/D velocity ratio was also among the top selected parameters by the ML models, despite being present in less than half of the patients. This parameter had the second highest accuracy as standalone parameter in the regression plots (see Supplementary data online, *Figure S1*). Thus, the ML selection of this ratio and proBNP-parameters, which are less commonly assessed but well correlated with LVFP, suggests that they should be evaluated in the classification when present. Body surface area and weight were also among the selected parameters, which may suggest that such phenotypical parameters can have value in the classification.

There may be reluctance against clinical use of ML based methods as many of them are ‘black box models’, meaning the algorithm and calculations to obtain the classification result are hidden from the user. However, the tree-based ML models used in this study are transparent and can be visualized. Potentially, software could be developed to provide a graphical interpretation of the path through the ML model tree with the deciding parameters of each patient. This could be particularly useful in cases where the model reaches a different conclusion than the clinician: disclosing the interpretation of the model, may in some cases give a clue to something the clinician may have overlooked, or in cases where the model has clearly not considered a confounding factor that the clinician is aware of, the model output can be ignored. In this manner, the model will serve as a diagnostic support system aiding the clinician, complimenting the conventional approach.

### Limitations

To mitigate the risk of overfitting and compensate for the absence of external validation, we employed nested cross-validation to evaluate model performance. While this approach provides a more robust estimate of generalization error compared to standard cross-validation, it cannot fully substitute for validation on an independent cohort. Hence, we cannot exclude that overfitting has occurred. While 250 patients may be considered a large dataset in traditional classification studies, it is a small dataset to develop ML-based methods. Several parameters carry information related to LVFP and disclosing their complex relationship requires large amounts of data. Therefore, the accuracies reported in this study should be considered an indication of the potential of such ML models. Potentially the performance could be better with more data.

The performance of the ML models, as well as the conventional algorithms and most single parameters, was poorer in patients with preserved EF. As patients with preserved EF differ in pathophysiology and clinical phenotype from those with reduced EF, performance of the ML models may be improved by developing separate models for these populations. However, that would have required more data in each group than we had available. The reported results for the different EF subgroups were based on relatively low sample sizes and should therefore be interpreted with caution.

The ML models selected on average 13 parameters. Practically no patients had measured values for all the selected parameters, which hence would leave a large number of patients unclassified. Only the XGBoost ML model was trained and tested on data with missing parameters as its algorithm automatically deals with missing values. In order to avoid unclassified patients for the seven other ML models, missing values were replaced with their cutoff values. This replacement strategy aimed to minimize the influence of missing parameter values on the classification result and performed slightly better than replacing with mean or median values (results not shown). However, such replacement strategies may disrupt the relationships between parameters, which could be important particularly in ML. Consequently, some inferior performance may have been introduced as a trade-off for classifying all patients.

The data in this study were acquired to compare with the 2016 ASE/EACVI recommendations for evaluation of diastolic function.^1^ The recently published 2025 ASE recommendations^14^ require additional parameters that were not measured in the present study. This includes estimate of right atrial pressure and isovolumic relaxation time, which were not available in the current dataset, and pulmonary venous S/D ratio which was missing in more than half the patients. A reliable evaluation of the new algorithm’s performance was not possible due to the absence of these parameters in the current dataset. Future studies should investigate whether ML approaches may further improve diagnostic accuracy in the assessment of LV filling pressure with the addition of the parameters recommended in the 2025 ASE guidelines.

## Conclusion

ML may improve classification of LVFP. The investigated ML models had comparable accuracy to the expert derived algorithm but had superior feasibility and left no patients unclassified. Because the ML models handle missing parameters, they can take advantage of rare, but good parameters when present. The ML models provided new insights into which parameters may be most valuable to use in the classification. These included parameters that are less commonly obtained, but well correlated with LVFP. The results encourage further development of ML based methods on more patient data that will presumably further improve accuracy of LVFP classification and lead to utilization of such ML models in clinical practice.

## Supplementary Material

jeag025_Supplementary_Data

## Data Availability

There is no approval from the different ethical committees of this multicentre study to publicly share the patient data of this article.

## References

[1] Nagueh SF, Smiseth OA, Appleton CP, Byrd BF III, Dokainish H, Edvardsen T et al Recommendations for the evaluation of left ventricular diastolic function by echocardiography: an update from the American Society of Echocardiography and the European Association of Cardiovascular Imaging. Eur Heart J Cardiovasc Imaging 2016;17:1321–60. 27422899 10.1093/ehjci/jew082

[2] Andersen OS, Smiseth OA, Dokainish H, Abudiab MM, Schutt RC, Kumar A et al Estimating left ventricular filling pressure by echocardiography. J Am Coll Cardiol 2017;69:1937–48. 28408024 10.1016/j.jacc.2017.01.058

[3] Lancellotti P, Galderisi M, Edvardsen T, Donal E, Goliasch G, Cardim N et al Echo-Doppler estimation of left ventricular filling pressure: results of the multicentre EACVI Euro-Filling study. Eur Heart J Cardiovasc Imaging 2017;18:961–8. 28444160 10.1093/ehjci/jex067

[4] Inoue K, Khan FH, Remme EW, Ohte N, García-Izquierdo E, Chetrit M et al Determinants of left atrial reservoir and pump strain and use of atrial strain for evaluation of left ventricular filling pressure. Eur Heart J Cardiovasc Imaging 2021;23:61–70. 33496314 10.1093/ehjci/jeaa415 PMC8685600

[5] Smiseth OA, Morris DA, Cardim N, Cikes M, Delgado V, Donal E et al Multimodality imaging in patients with heart failure and preserved ejection fraction: an expert consensus document of the European Association of Cardiovascular Imaging. Eur Heart J Cardiovasc Imaging 2022;23:e34–61. 34729586 10.1093/ehjci/jeab154

[6] Pandey A, Kagiyama N, Yanamala N, Segar MW, Cho JS, Tokodi M et al Deep-learning models for the echocardiographic assessment of diastolic dysfunction. JACC Cardiovasc Imaging 2021;14:1887–900. 34023263 10.1016/j.jcmg.2021.04.010

[7] Braunauer K, Düngen HD, Belyavskiy E, Aravind-Kumar R, Frydas A, Kropf M et al Potential usefulness and clinical relevance of a novel left atrial filling index to estimate left ventricular filling pressures in patients with preserved left ventricular ejection fraction. Eur Heart J Cardiovasc Imaging 2020;21:260–9. 31740950 10.1093/ehjci/jez272

[8] Smiseth OA, Fernandes JF, Ohte N, Wakami K, Donal E, Remme EW et al Imaging-based method to quantify left ventricular diastolic pressures. Eur Heart J Cardiovasc Imaging 2025;26:1184–94. 39821267 10.1093/ehjci/jeaf017 PMC12206579

[9] Gruca MM, Slivnick JA, Singh A, Cotella JI, Subashchandran V, Prabhu D et al Noninvasive assessment of left ventricular end-diastolic pressure using machine learning-derived phasic left atrial strain. Eur Heart J Cardiovasc Imaging 2023;25:18–26. 37708373 10.1093/ehjci/jead231

[10] Carluccio E, Cameli M, Rossi A, Dini FL, Biagioli P, Mengoni A et al Left atrial strain in the assessment of diastolic function in heart failure: a machine learning approach. Circ Cardiovasc Imaging 2023;16:e014605. 36752112 10.1161/CIRCIMAGING.122.014605

[11] Kim M, Bae S, Park JH, Jung IH. Relative importance of left atrial reservoir strain compared with components of the HFA-PEFF score: a cross-sectional study. Front Cardiovasc Med 2023;10:1213557. 37900564 10.3389/fcvm.2023.1213557 PMC10602785

[12] Remme EW, Inoue K, Smiseth OA. Machine learning in diastolic dysfunction: left atrial strain trace superior to single points for estimation of filling pressure. Eur Heart J Cardiovasc Imaging 2023;25:27–8. 37818845 10.1093/ehjci/jead257 PMC10735308

[13] Badano LP, Kolias TJ, Muraru D, Abraham TP, Aurigemma G, Edvardsen T et al Standardization of left atrial, right ventricular, and right atrial deformation imaging using two-dimensional speckle tracking echocardiography: a consensus document of the EACVI/ASE/Industry Task Force to standardize deformation imaging. Eur Heart J Cardiovasc Imaging 2018;19:591–600. 29596561 10.1093/ehjci/jey042

[14] Nagueh SF, Sanborn DY, Oh JK, Anderson B, Billick K, Derumeaux G et al Recommendations for the evaluation of left ventricular diastolic function by echocardiography and for heart failure with preserved ejection fraction diagnosis: an update from the American society of echocardiography. J Am Soc Echocardiogr 2025;38:537–69. 40617625 10.1016/j.echo.2025.03.011

