# Supplementary material for: Evaluation of diastolic function: machine learning improves classification of left ventricular filling pressure
Source: Eur Heart J Cardiovasc Imaging. 2026 Jan 28;27(6):1205–12. doi: 10.1093/ehjci/jeag025 (PMC13222719; doi:10.1093/ehjci/jeag025)
Supplement: jeag025_Supplementary_Data [file jeag025_supplementary_data.docx]

**Supplemental Material**

**to the paper**

**“Evaluation of diastolic function: Machine learning improves classification of left ventricular filling pressure”**

**Figure S1** - Regression plots between left ventricular (LV) filling pressure and the 64 parameters in all patients, page 2-13.

**Supplemental Methods** - pages 14-15.

**Tables S1-S4** - Performance of the different machine learning models, the 2016 ASE/EACVI algorithm, the 2022 EACVI algorithm and single parameters, pages 16-19.

**Figure S1.** Regression plots between left ventricular (LV) filling pressure and the 64 parameters that were initially evaluated for use in the machine learning models. Not all parameters were obtained in all 250 patients. The number of patients, N, with each parameter is shown. The optimal cutoff value to differentiate between normal and elevated LV filling pressure for each parameter, is shown, which was found using logistic regression. In some plots the cutoff was outside the parameter value range, which means that classifying all patients as either having normal or elevated, provided the best accuracy in these cases. The accuracy and receiver operating area under the curve (AUC) values for each parameter as a stand-alone index are included. The plots are sorted and numbered in descending order of accuracy. AR = atrial reversal; ED = end diastolic; ES = end systolic; LA = left atrial; LAVI = LA volume index; LV = left ventricular; PV = pulmonary vein; TR = tricuspid regurgitation.


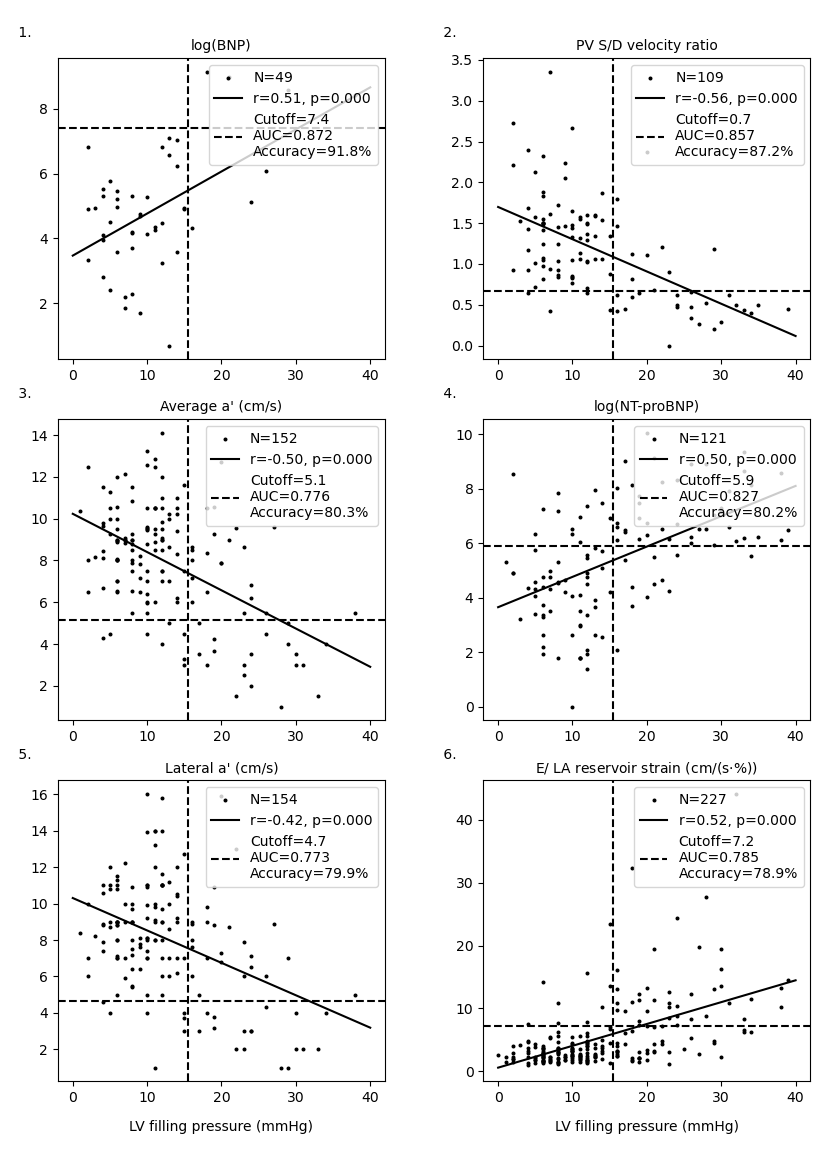

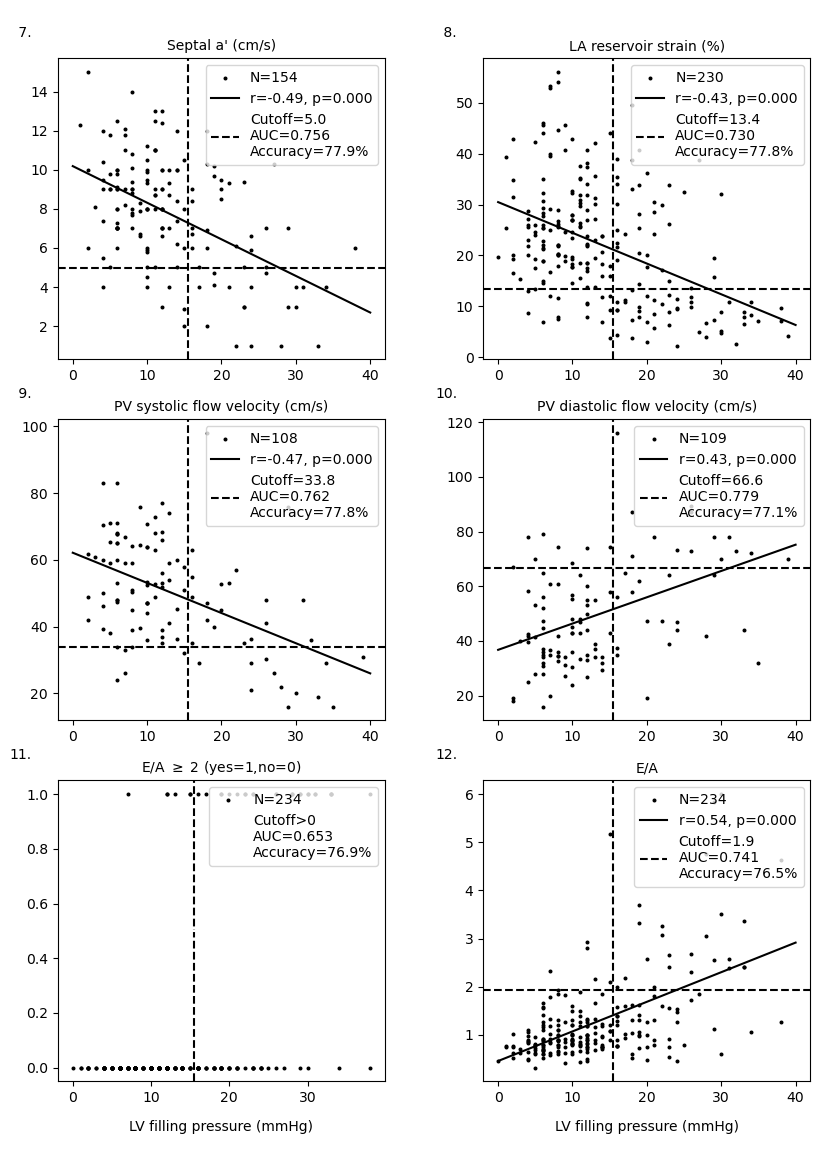

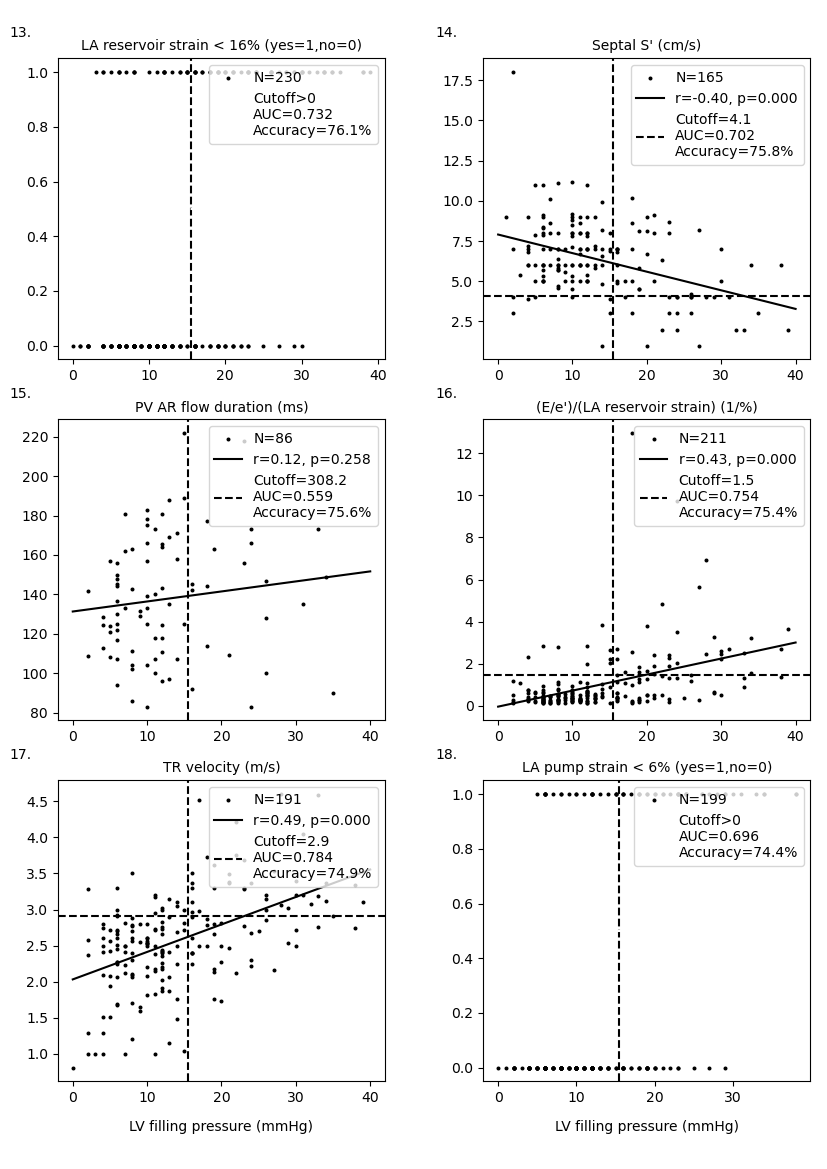

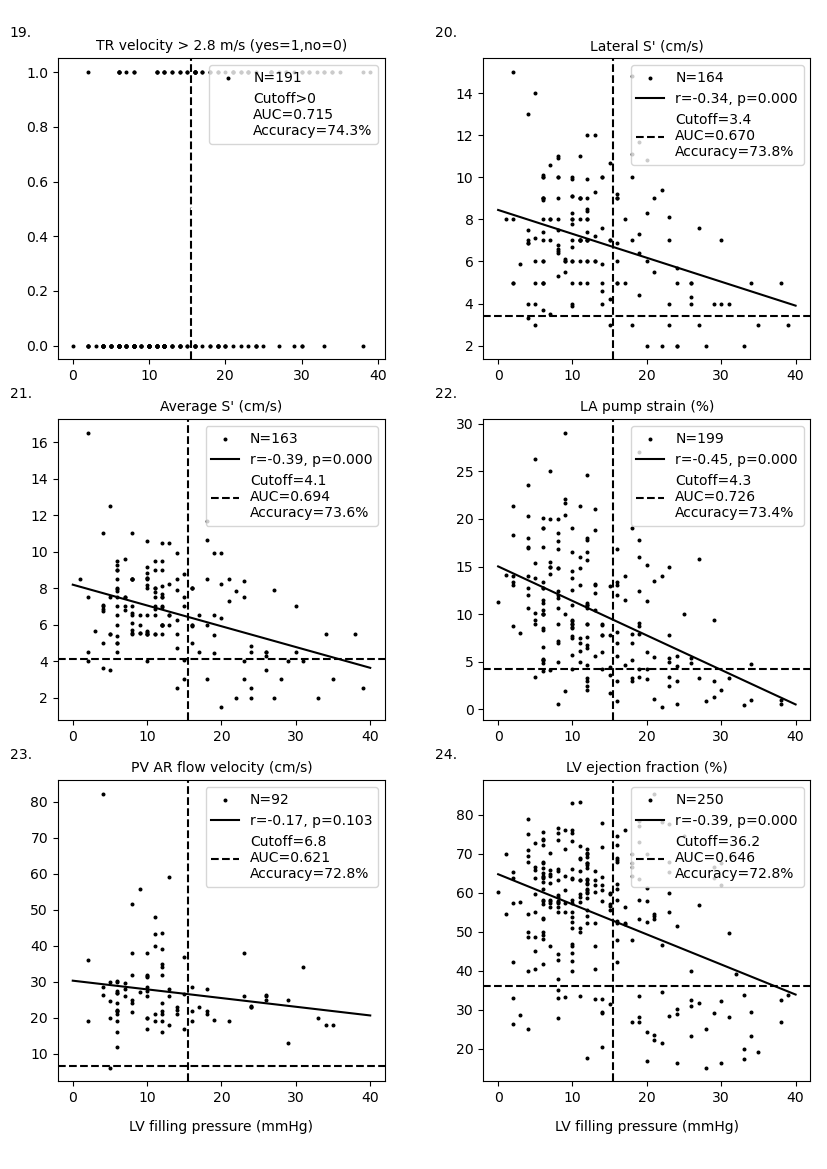

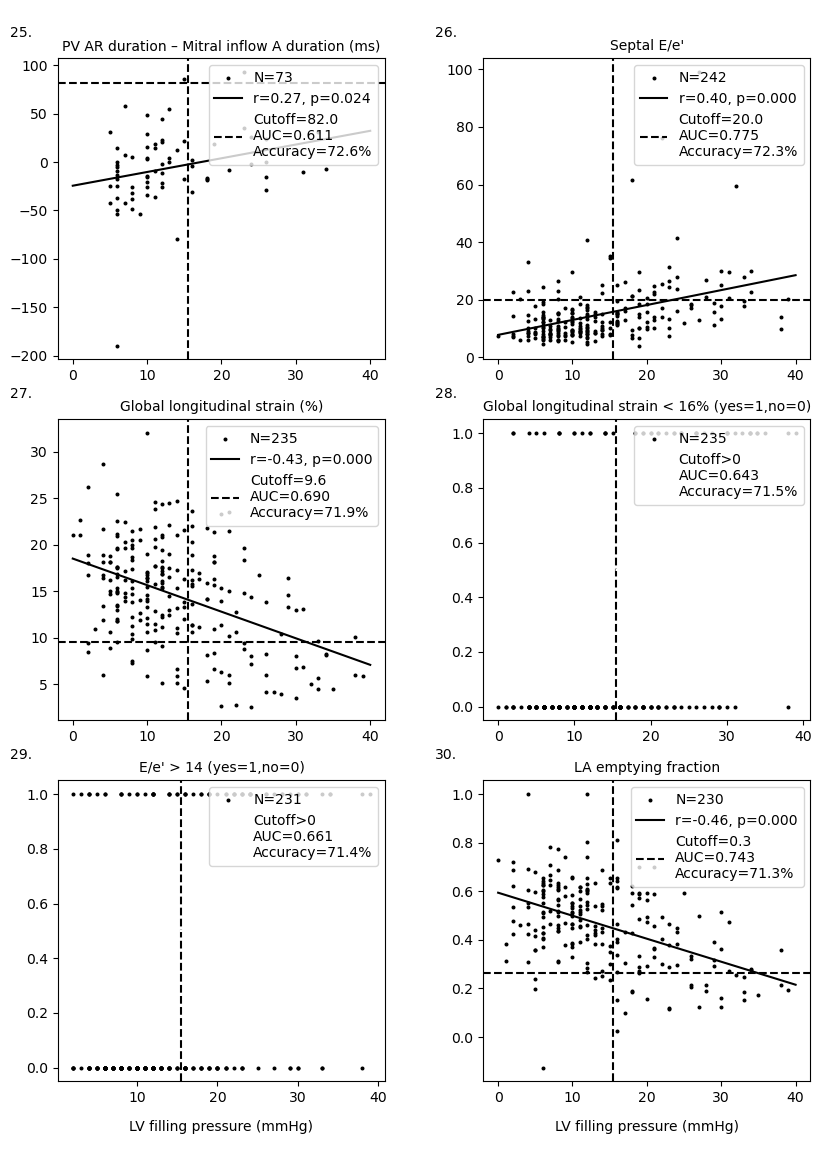

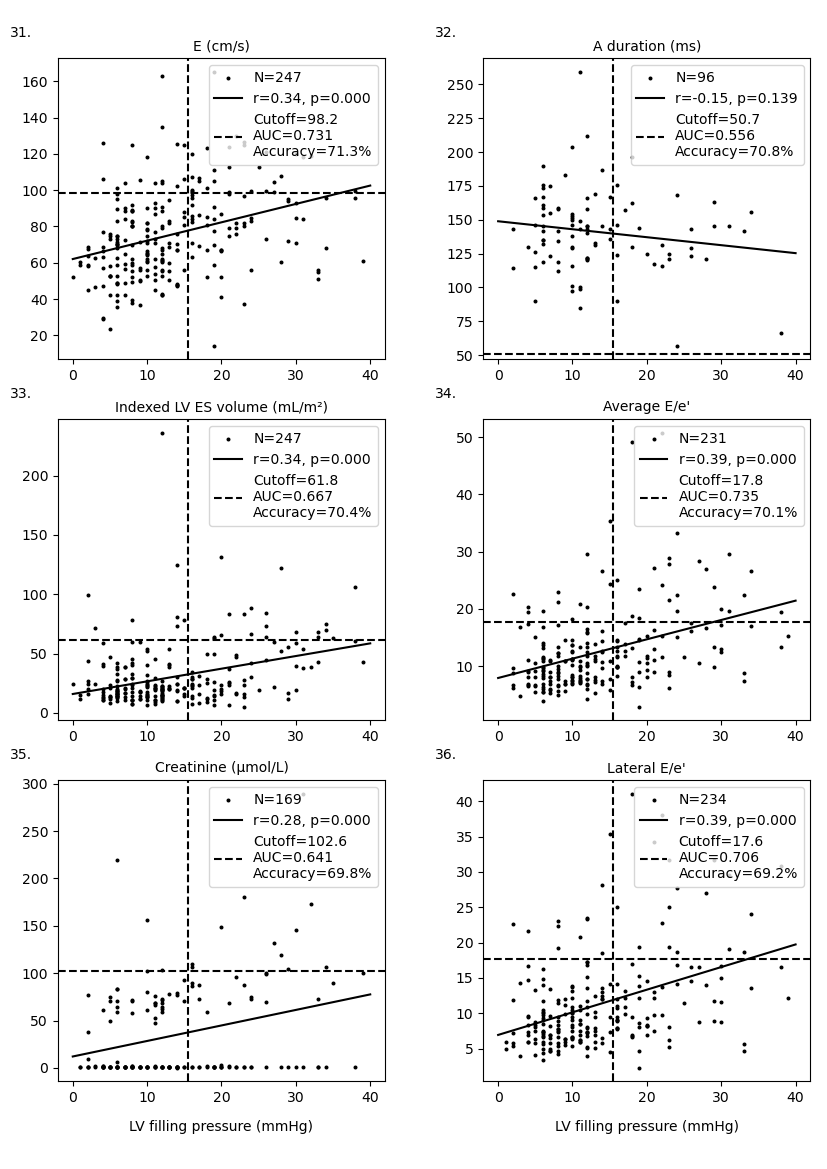

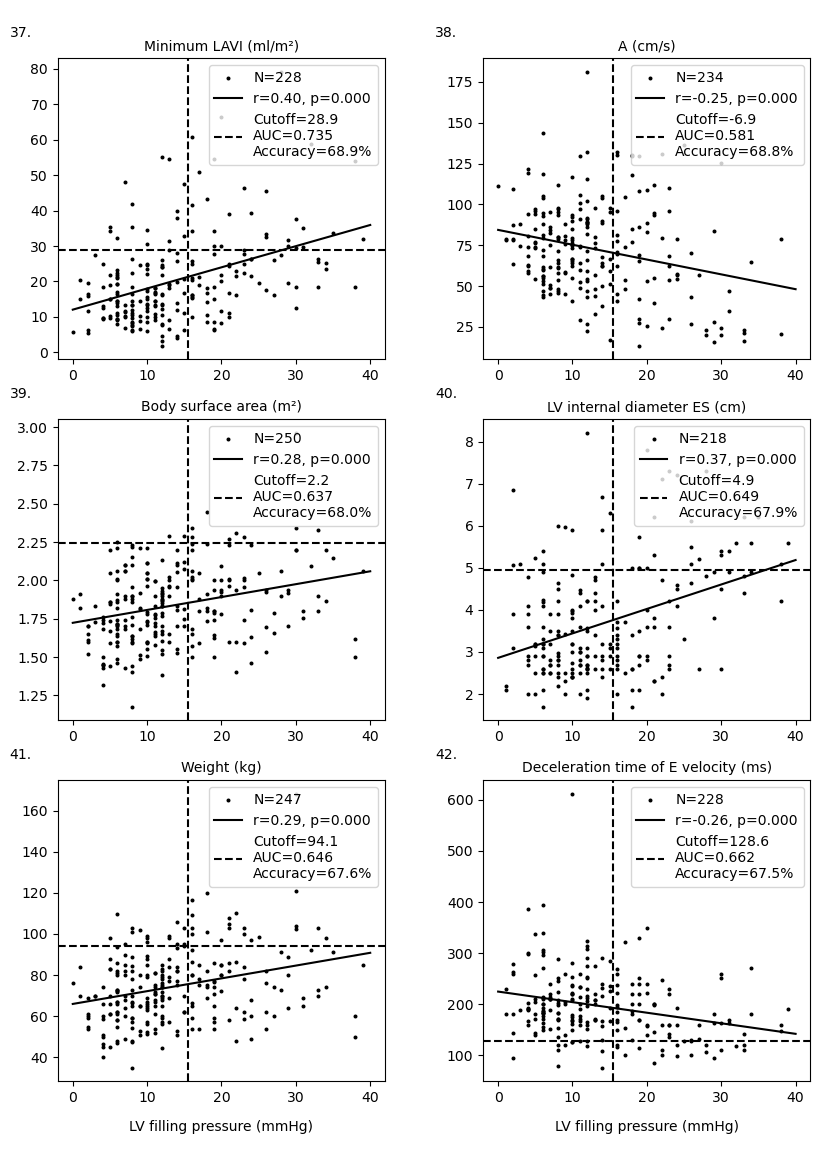

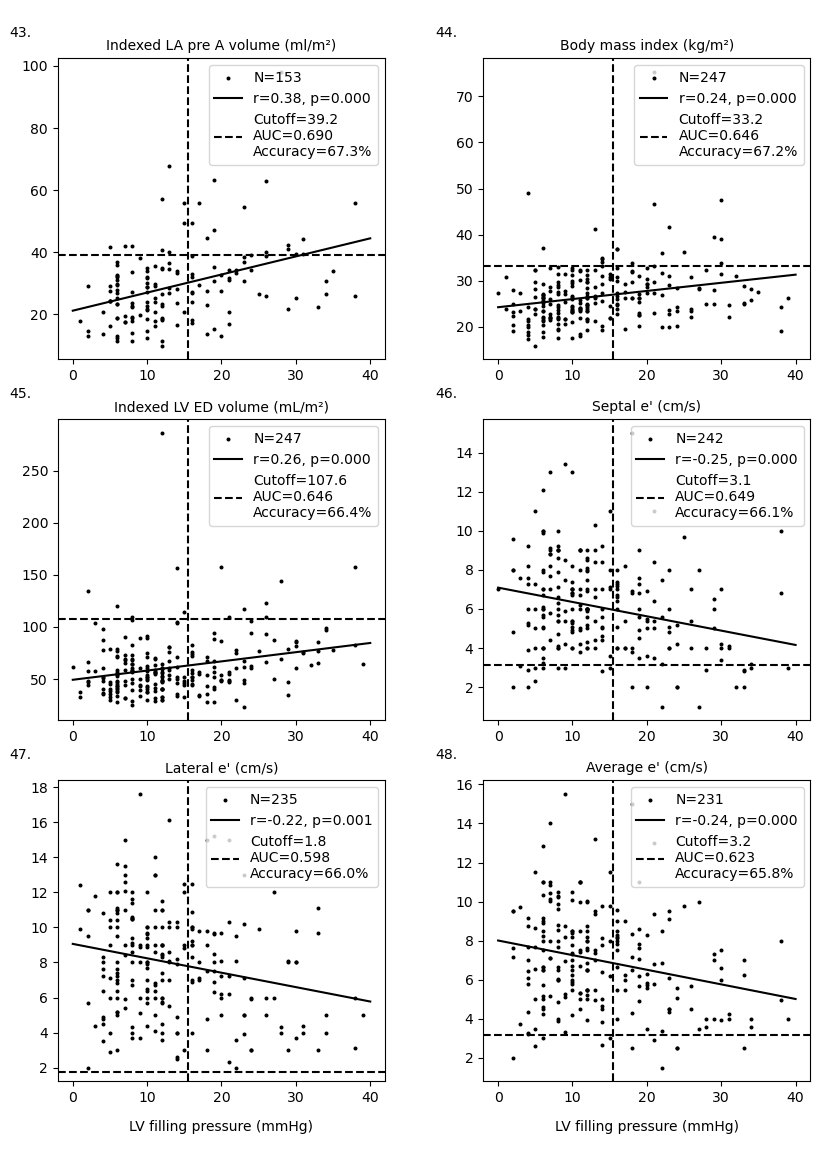

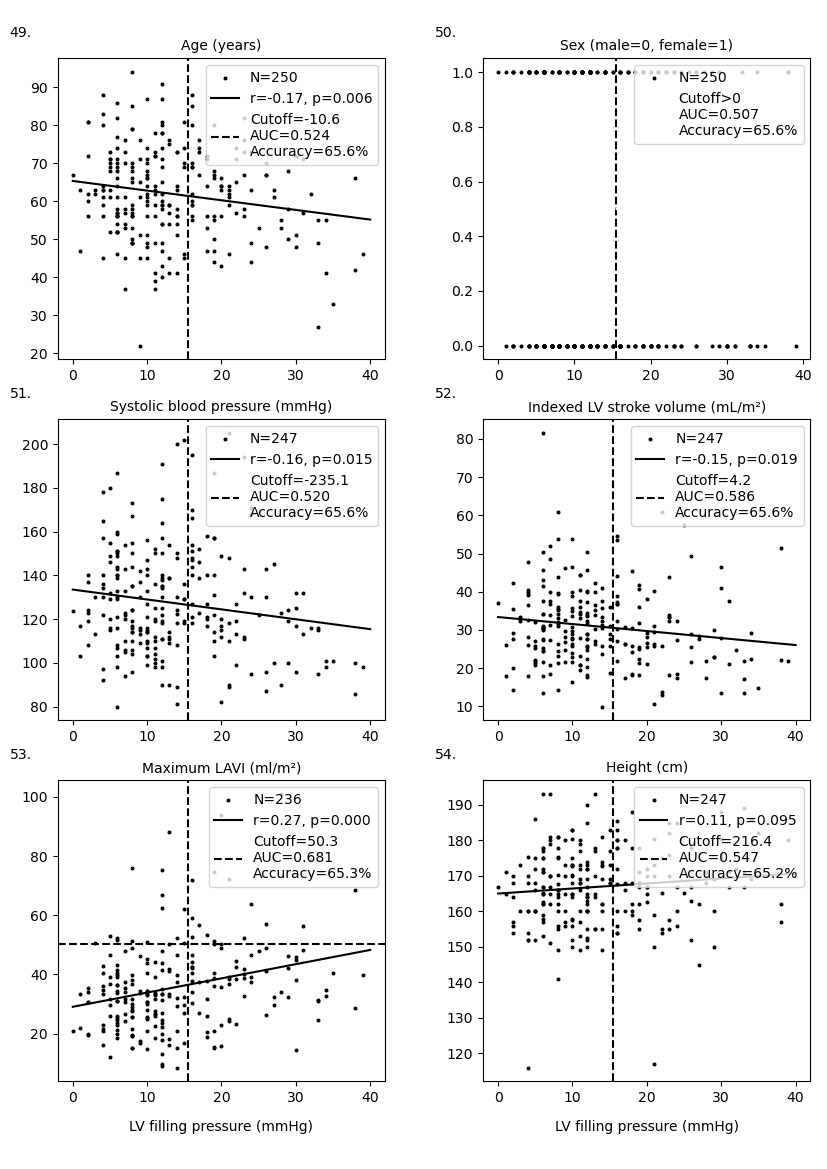

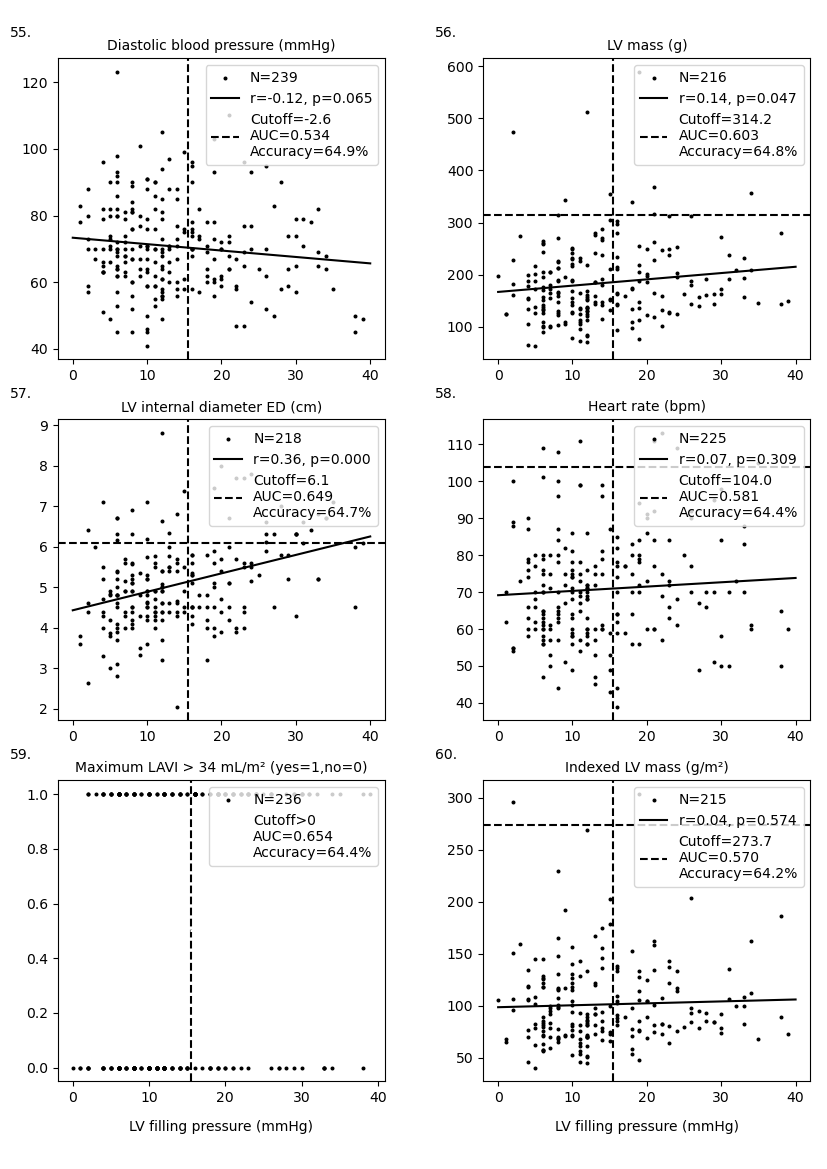

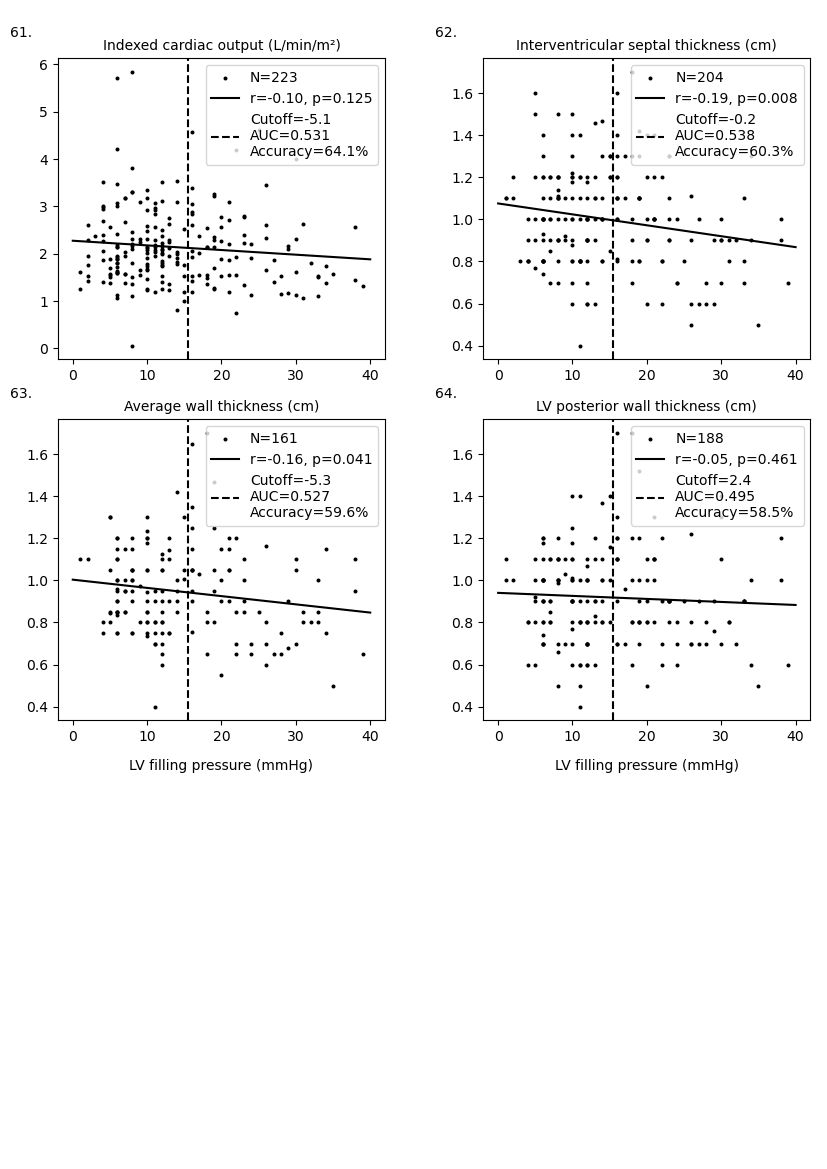


**Supplemental methods**

In this study, we employed the eight machine learning classifiers from the XGBoost library (version 2.0.3) and scikit-learn library (version 1.3.0) as listed below together with their hyperparameters and the search ranges for these during training of each model.

Extreme Gradient Boosting classifier (XGB)

The XGB classifier is a powerful machine learning algorithm that uses an ensemble of decision trees to improve predictive accuracy. A full grid search was performed for the hyperparameters over the values in brackets: n_estimators [20, 25, 30, 35, 40], max_depth [1, 2], and learning_rate [0.01, 0.1, 0.2, 0.3, 0.4], gamma [0, 0.5, 1, 3, 5], min_child_weight [1, 3 , 5, 7, 9]. The other hyperparameters were set to default values including the other regularization parameters reg_alpha and reg_lambda except tree_method=exact.

Support Vector Classifier (SVC)

SVC is a supervised learning algorithm that finds the optimal hyperplane to separate different classes in a dataset by maximizing the margin between the closest points of the classes. A full grid search was performed for the hyperparameters over the values in brackets: C [0.1, 0.25, 0.5, 1, 2, 3], kernel [linear, poly, rbf, sigmoid], degree [1,2,3], gamma [ 0.001, 0.01, 0.05, 0.1, 0.5]. Default values were used for the other hyperparameters except probability=True.

Linear Support Vector Classifier (LSVC)

LSVC is a supervised learning algorithm that finds the optimal linear hyperplane to separate different classes in a dataset by maximizing the margin between the closest points of the classes. A full grid search was performed for the hyperparameter over the values in brackets: C: [0.1, 0.25, 0.5, 1, 2, 3]. Default values were used for the other hyperparameters.

Gradient Boosting Classifier (GBC)

GBC is an ensemble learning algorithm that builds a series of weak learners, (i.e. decision trees), in a sequential manner, where each new model corrects the errors of the previous ones to improve overall prediction accuracy. A full grid search was performed for the hyperparameters over the values in brackets: n_estimators [20, 40, 60, 80, 100], max_depth [1,2,3], learning_rate [0.01, 0.05, 0.1, 0.25, 0.5]. Default values were used for the other hyperparameters.

Logistic Regression classifier (LR)

Logistic Regression is a supervised learning algorithm used for binary classification that models the probability of a class label by fitting a logistic function to the input parameters.

A full grid search was performed for the hyperparameter over the values in brackets: C [0.01, 0.05, 0.1, 0.5, 1, 2, 3, 4, 5]. Default values were used for the other hyperparameters except solver=liblinear.

K-Nearest Neighbours classifier (KNN)

KNN is a supervised learning algorithm that classifies a data point based on the majority class of its k-nearest neighbours in the parameter space. A full grid search was performed for the hyperparameter n_neighbors from 3 to 50. Default values were used for the other hyperparameters.

Random Forest classifier (RF)

RF is an ensemble learning algorithm that constructs multiple decision trees during training and outputs the mode of the classes of the individual trees for improved accuracy and robustness. A full grid search was performed for the hyperparameters over the values in brackets: n_estimators [20,40,60,80,100] and max_depth [1,2,3]. Default values were used for the other hyperparameters except max_features=1.0 and min_impurity_decrease=0.01.

Decision Tree classifier (DT)

DT is a supervised learning algorithm that splits the data into subsets based on the value of input parameters, creating a tree-like model of decisions to predict the class labels. A full grid search was performed for the hyperparameters over the values in brackets: max_depth [2,3,4,5], min_samples_leaf [3, 4, 5, 6, 7] and min_impurity_decrease [0.005, 0.01, 0.02]. Default values were used for the other hyperparameters.

**Data scaling**

For the classifiers SVC, LSVC, KNN, and LR, the data was scaled using Scikit-learn's StandardScaler, which transforms the dataset so that each parameter has a mean of 0 and a standard deviation of 1. The scaling factors were computed on the training set and then applied to both the training and test sets.

**Parameter importances**

We used the recursive feature elimination cross validation method (RFECV, scikit-learn 1.3.0) to find the selected parameters of all ML-models except for KNN, for which this method is not applicable. For KNN we employed the sequential feature selector method (SFS, mlxtend, 0.23.1). The parameter importances for the tree-based ML models XGB, SGB, RF and DT were ranked using each model’s default parameter importance scoring method: ‘gain’ for XGB and ‘mean decrease in impurity’ for the three others.

**Abbreviations to Tables S1-S4.**

Machine learning models:

XGB = Extreme Gradient Boosting classifier

SVC = Support Vector Classifier

LSVC = Linear Support Vector Classifier

GBC = Gradient Boosting Classifier

LR = Logistic Regression classifier

KNN = K-Nearest Neighbours classifier

RF = Random Forest classifier

DT = Decision Tree classifier

Additional abbreviations:

ASE = American society of echocardiography; AUC = area under the curve; EACVI = European association of cardiovascular imaging; EF = ejection fraction; LARS = left atrial reservoir strain; LAVI = maximum left atrial volume index; NPV = negative predictive value; PPV = positive predictive value; TRV = tricuspid regurgitation velocity.

**Table S1.** Performance of the different machine learning models on all 250 patients. There were no unclassified patients.

|  | Accuracy  (%) | AUC | Sensitivity  (%) | Specificity  (%) | PPV  (%) | NPV  (%) |
| --- | --- | --- | --- | --- | --- | --- |
| SVC | 84 | 0.87 | 65 | 94 | 86 | 84 |
| LSVC | 84 | 0.89 | 71 | 92 | 82 | 86 |
| XGB | 82 | 0.89 | 69 | 89 | 79 | 85 |
| SGB | 83 | 0.89 | 68 | 91 | 81 | 84 |
| LR | 86 | 0.87 | 75 | 92 | 84 | 87 |
| KNN | 82 | 0.84 | 55 | 96 | 87 | 80 |
| RF | 83 | 0.86 | 61 | 95 | 86 | 82 |
| DT | 84 | 0.80 | 66 | 93 | 83 | 84 |

**Table S2.** Performance of the different machine learning models, the 2016 ASE/EACVI algorithm, the 2022 EACVI algorithm and single parameters in the 173 patients with EF≥50% where 34.4% had elevated left ventricular filling pressure.

|  | Accuracy  (%) | AUC | Sensitivity  (%) | Specificity  (%) | PPV  (%) | NPV  (%) | Unclassified  (%) |
| --- | --- | --- | --- | --- | --- | --- | --- |
| SVC | 80 | 0.75 | 40 | 95 | 72 | 82 | 0 |
| LSVC | 80 | 0.80 | 49 | 91 | 67 | 84 | 0 |
| XGB | 78 | 0.80 | 44 | 90 | 61 | 82 | 0 |
| SGB | 80 | 0.79 | 42 | 93 | 68 | 82 | 0 |
| LR | 83 | 0.77 | 53 | 93 | 73 | 85 | 0 |
| KNN | 78 | 0.72 | 22 | 98 | 77 | 78 | 0 |
| RF | 80 | 0.74 | 33 | 96 | 75 | 80 | 0 |
| DT | 82 | 0.66 | 47 | 94 | 72 | 83 | 0 |
| 2016 ASE/EACVI | 80 | 0.73 | 59 | 87 | 61 | 86 | 15 |
| 2022 EACVI | 77 | 0.68 | 50 | 86 | 56 | 83 | 4 |
| E/e’>14 | 73 | 0.59 | 30 | 88 | 48 | 78 | 6 |
| TRV>2.8m/s | 72 | 0.64 | 45 | 82 | 48 | 80 | 30 |
| LAVI>34mL/m² | 66 | 0.66 | 65 | 67 | 42 | 84 | 8 |
| E/A<0.8 | 42 | 0.53 | 75 | 30 | 27 | 78 | 1 |
| E/A>2 | 76 | 0.55 | 11 | 98 | 63 | 76 | 1 |
| LARS<23% | 57 | 0.54 | 48 | 60 | 32 | 74 | 10 |
| LARS<18% | 70 | 0.60 | 36 | 84 | 47 | 77 | 10 |
| E/LARS>3 | 63 | 0.64 | 66 | 61 | 40 | 82 | 10 |
| E/LARS>5 | 74 | 0.62 | 34 | 89 | 56 | 77 | 10 |

**Table S3.** Performance of the different machine learning models, the 2016 ASE/EACVI algorithm, the 2022 EACVI algorithm and single parameters in the 77 patients with EF<50% where 53.2% had elevated left ventricular filling pressure.

|  | Accuracy  (%) | AUC | Sensitivity  (%) | Specificity  (%) | PPV  (%) | NPV  (%) | Unclassified  (%) |
| --- | --- | --- | --- | --- | --- | --- | --- |
| SVC | 92 | 0.97 | 93 | 92 | 93 | 92 | 0 |
| LSVC | 94 | 0.93 | 95 | 92 | 93 | 94 | 0 |
| XGB | 91 | 0.94 | 95 | 86 | 89 | 94 | 0 |
| SGB | 90 | 0.92 | 95 | 83 | 87 | 94 | 0 |
| LR | 94 | 0.96 | 98 | 89 | 91 | 97 | 0 |
| KNN | 90 | 0.92 | 90 | 89 | 90 | 89 | 0 |
| RF | 91 | 0.93 | 90 | 92 | 93 | 89 | 0 |
| DT | 88 | 0.90 | 88 | 89 | 90 | 86 | 0 |
| 2016 ASE/EACVI | 83 | 0.82 | 88 | 77 | 83 | 83 | 8 |
| 2022 EACVI | 82 | 0.81 | 88 | 75 | 80 | 84 | 0 |
| E/e’>14 | 68 | 0.68 | 70 | 66 | 70 | 66 | 10 |
| TRV>2.8m/s | 79 | 0.79 | 72 | 87 | 88 | 71 | 9 |
| LAVI>34mL/m² | 61 | 0.59 | 73 | 46 | 61 | 59 | 1 |
| E/A<0.8 | 66 | 0.68 | 93 | 42 | 59 | 88 | 19 |
| E/A>2 | 77 | 0.76 | 62 | 91 | 86 | 73 | 19 |
| LARS<23% | 69 | 0.68 | 100 | 36 | 63 | 100 | 3 |
| LARS<18% | 79 | 0.78 | 97 | 58 | 72 | 95 | 3 |
| E/LARS>3 | 74 | 0.73 | 100 | 46 | 66 | 100 | 6 |
| E/LARS>5 | 86 | 0.86 | 92 | 80 | 83 | 90 | 6 |

**Table S4.** Performance of the different machine learning models, the 2016 ASE/EACVI algorithm, the 2022 EACVI algorithm and single parameters in the 52 patients with EF<40% where 67.3% had elevated left ventricular filling pressure.

|  | Accuracy  (%) | AUC | Sensitivity  (%) | Specificity  (%) | PPV  (%) | NPV  (%) | Unclassified  (%) |
| --- | --- | --- | --- | --- | --- | --- | --- |
| SVC | 90 | 0.96 | 94 | 82 | 92 | 88 | 0 |
| LSVC | 90 | 0.87 | 94 | 82 | 92 | 88 | 0 |
| XGB | 87 | 0.88 | 94 | 71 | 87 | 86 | 0 |
| SGB | 87 | 0.84 | 94 | 71 | 87 | 86 | 0 |
| LR | 92 | 0.92 | 97 | 82 | 92 | 93 | 0 |
| KNN | 88 | 0.86 | 91 | 82 | 91 | 82 | 0 |
| RF | 87 | 0.86 | 89 | 82 | 91 | 78 | 0 |
| DT | 85 | 0.80 | 89 | 76 | 89 | 76 | 0 |
| 2016 ASE/EACVI | 82 | 0.79 | 88 | 71 | 86 | 75 | 2 |
| 2022 EACVI | 83 | 0.80 | 89 | 71 | 86 | 75 | 0 |
| E/e’>14 | 61 | 0.57 | 68 | 47 | 72 | 41 | 12 |
| TRV>2.8m/s | 78 | 0.82 | 70 | 94 | 96 | 60 | 6 |
| LAVI>34mL/m² | 63 | 0.58 | 74 | 41 | 72 | 44 | 0 |
| E/A<0.8 | 79 | 0.73 | 96 | 50 | 77 | 88 | 25 |
| E/A>2 | 72 | 0.75 | 64 | 86 | 89 | 57 | 25 |
| LARS<23% | 72 | 0.59 | 100 | 18 | 70 | 100 | 4 |
| LARS<18% | 80 | 0.71 | 100 | 41 | 77 | 100 | 4 |
| E/LARS>3 | 81 | 0.72 | 100 | 44 | 78 | 100 | 10 |
| E/LARS>5 | 87 | 0.83 | 97 | 69 | 86 | 92 | 10 |
